# Wood fibers are a crucial microhabitat for cellulose- and xylan- degrading bacteria in the hindgut of the wood-feeding beetle *Odontotaenius disjunctus*

**DOI:** 10.3389/fmicb.2023.1173696

**Published:** 2023-06-28

**Authors:** Melbert Schwarz, Cristian F. Beza-Beza, Aram Mikaelyan

**Affiliations:** Department of Entomology and Plant Pathology, North Carolina State University, Raleigh, NC, United States

**Keywords:** beetles, Passalidae, lignocellulose, symbiotic digestion, gut microbiomes

## Abstract

**Introduction:**

Wood digestion in insects relies on the maintenance of a mosaic of numerous microhabitats, each colonized by distinct microbiomes. Understanding the division of digestive labor between these microhabitats- is central to understanding the physiology and evolution of symbiotic wood digestion. A microhabitat that has emerged to be of direct relevance to the process of lignocellulose digestion is the surface of ingested plant material. Wood particles in the guts of some termites are colonized by a specialized bacterial fiber-digesting microbiome, but whether this represents a widespread strategy among insect lineages that have independently evolved wood-feeding remains an open question.

**Methods:**

In this study, we investigated the bacterial communities specifically associated with wood fibers in the gut of the passalid beetle *Odontotaenius disjunctus*. We developed a Percoll-based centrifugation method to isolate and enrich the wood particles from the anterior hindgut, allowing us to access the wood fibers and their associated microbiome. We then performed assays of enzyme activity and used short-read and long-read amplicon sequencing of the 16S rRNA gene to identify the composition of the fiber-associated microbiome.

**Results:**

Our assays demonstrated that the anterior hindgut, which houses a majority of the bacterial load, is an important site for lignocellulose digestion. Wood particles enriched from the anterior hindgut contribute to a large proportion of the total enzyme activity. The sequencing revealed that *O. disjunctus*, like termites, harbors a distinct fiber-associated microbiome, but notably, its community is enriched in insect-specific groups of *Lactococcus* and *Turicibacter*.

**Discussion:**

Our study underscores the importance of microhabitats in fostering the complex symbiotic relationships between wood-feeding insects and their microbiomes. The discovery of distinct fiber-digesting symbionts in *O. disjunctus*, compared to termites, highlights the diverse evolutionary paths insects have taken to adapt to a challenging diet.

## 1. Introduction

The recycling of wood is central to terrestrial ecosystems, and the process critically depends on the activities of wood-feeding insects (Ulyshen and Wagner, 2013). Wood, composed primarily of lignocellulose, is both recalcitrant and lacking in fixed nitrogen (Ulyshen, 2018), thus making it a poor resource for most animals. Despite the many dietary challenges posed by wood, the ability to utilize it has independently evolved multiple times among insects, most notably among the termites, panesthiine cockroaches, and passalid beetles (Watanabe and Tokuda, 2010). Because insects synthesize only a limited repertoire of enzymes required for efficient lignocellulose digestion, all described wood-feeding species rely on intricate symbiotic mechanisms for the breakdown of wood, often involving members of the gut microbiome (Watanabe and Tokuda, 2010).

Most of the knowledge on symbiotic wood digestion is derived from literature on termites, and while other wood-feeding insects are likely to differ in some key steps of the process, many generalities hold true. Digestion starts with the physical breakdown of wood through the action of sclerotized mandibles; enzymes predominantly synthesized by the insect in the foregut or midgut then partially digest the wood particles, as they flow into an enlarged hindgut (Watanabe and Tokuda, 2010). Although only a few microliters in volume, the hindguts of all known wood-feeding insects possess highly heterogeneous environments (Brune and Dietrich, 2015; Ceja-Navarro et al., 2019). Moreover, they are defined by steep variations in physicochemical parameters that partition the microbiome into distinct microhabitats (Brune et al., 1995; Yang et al., 2005; Köhler et al., 2012; Bauer et al., 2015; Mikaelyan et al., 2017; Ceja-Navarro et al., 2019). This inherently complex nature of symbiotic wood digestion impresses a need to better integrate our understanding of microbial ecology across different spatial scales with regards to the digestive physiology of wood feeders. One approach to addressing such important questions is to explore this process in insect lineages which have convergently evolved the capacity to feed on wood.

Wood-boring beetles of the family Passalidae offer an ideal opportunity to study a symbiotic mechanism that has evolved independently to surmount digestive challenges similar to those encountered by termites. The horned passalus *Odontotaenius disjunctus* is a widely distributed passalid species in North America and has long been studied from the perspective of the gut microbiome (Leidy, 1849, 1861; Pearse et al., 1936; Suh et al., 2003; Nardi et al., 2006; Urbina et al., 2013). The remarkable microheterogeneity of the gut ecosystem of *O. disjunctus* (Ceja-Navarro et al., 2014, 2019) is similar to that of termites (Brune, 2014), and supports a comparably complex, yet distinct gut microbiome. A recent omics-based study in this beetle species (Ceja-Navarro et al., 2019) has also highlighted the potential role of certain members of its hindgut microbiome in the digestion of lignocellulose. However, we face two major knowledge gaps in our understanding of wood digestion in passalid beetles. First, in the absence of quantitative measurements of enzyme activity associated with lignocellulose digestion, the true partitioning of the digestive capabilities of passalids remains unclear. Second, a critical component in the link between microbiome structure and function is to interrogate microbiome composition at the appropriate spatial scale, which has yet to be elucidated in these beetles. In the context of anaerobic lignocellulose digestion in gut ecosystems, the surfaces of wood particles act as key microhabitats for specialized bacterial communities. While fiber-associated communities have indeed been shown to be central to symbiotic wood digestion in termites (Mikaelyan et al., 2014; Tokuda et al., 2018), it is not clear if they are central to wood digestion in other insects as well.

In this study, we attempt to address these relevant and important knowledge gaps by comprehensively analyzing the microbial ecology of wood digestion in passalid beetles. Specifically, our aim was to elucidate the contribution of bacteria to lignocellulose digestion. We directly quantified the cellulase and xylanase activities along the gut axis of *O. disjunctus*. Using quantitative PCR (qPCR) of the 16S rRNA gene, in conjunction with quantification of enzymatic activity, we identified the anterior hindgut (AHG) of the beetles as a potentially important site within the gut for bacterial lignocellulose digestion. Based on this convincing evidence, we further investigated the enzymatic activity associated with microhabitats in the AHG. We enriched wood particles from the AHG lumen, along with any associated bacteria, using a modification of an established protocol (Mikaelyan et al., 2014). Both cellulase and xylanase activity associated with the fiber fraction and its planktonic (i.e., cells free-floating within the lumen) counterpart was quantified. In addition, we further characterized the fiber-associated and planktonic communities by Illumina-sequencing the amplified V3-V4 region of 16S rRNA genes. Finally, using longer 16S rRNA sequences generated with the PacBio platform, we investigated the phylogenetic position of the bacterial lineages associated with wood fibers in the AHG. By further elucidating the microhabitats associated with symbiotic wood digestion in *O. disjunctus*, we sought to better understand the common mechanisms for lignocellulose digestion it shares with other wood-feeding insects such as termites.

## 2. Materials and methods

### 2.1. *O. disjunctus*, maintenance, and dissection

*Odontotaenius disjunctus* adults were acquired from a commercial supplier (Carolina Biological, NC, USA), and were kept in a plastic container with potting soil (Nature’s care Organic Potting soil; Miracle grow), decaying oak wood and sphagnum moss for 4 weeks prior to sampling. The container was stored in a climate-controlled room with a 12 h: 12 h light dark cycle, room temperature of 30°C, and a relative humidity of 80%. Moisture in the containers was maintained by spraying water every other day. Prior to dissection, adult *O. disjunctus* were immobilized by chilling at 4°C for 30 min and surface sterilized with 70% ethanol on an autoclaved silicone dissection pad. Their guts were harvested and the midgut (MG), anterior hindgut (AHG), and posterior hindgut (PHG) were recovered for downstream processing. Our study focused only on the midgut and hindgut regions, and in the rest of the article, we will refer to the MG, AHG, and PHG as compartments.

### 2.2. Preparation of fiber and fiber-free fractions from the AHG

To extract luminal contents, the MG, AHG, and PHG of *O. disjunctus* were split longitudinally using a sterile razor blade. The luminal content was gently mixed with 1X phosphate buffered saline (PBS; pH 7.0) and transferred into sterile 2.0 ml microcentrifuge tubes. Wood fibers from the AHG were enriched using the method stablished in Mikaelyan et al. (2014) with minor modifications. A stock solution of 1X Percoll (GE Life Sciences), prepared in sterile 1X PBS, was filter-sterilized using a 0.22 μm syringe filter (Genesee Scientific). It was further diluted to obtain working solutions with 70 and 30% Percoll. In a sterile 2.0 ml microcentrifuge tube, a discontinuous density column was prepared by gently layering 900 μl of the 30% Percoll solution over 900 μl of the 70% solution. Pooled luminal content (∼ 100 μl) from two beetles was gently layered over the Percoll gradient and centrifuged (Microfuge 20R; FA241.5P rotor; Beckman Coulter) at 20,000 × *g* and 4°C for 30 min. This process yielded two well-separated bands across the Percoll solution. The formation of a gradient profile was verified in a separate control column using density marker beads (Cospheric; Supplementary Figure 1). The opaque layer of cells at the top, and the brown-colored layer containing wood particles at the bottom of the column, hereafter referred to as the “fiber-free” and “fiber” fractions, respectively, were transferred via micropipette to fresh tubes. Upon microscopic investigation, the middle layer (representing ∼1.3 ml) of the column was found to only contain very few or no particles or cells and was therefore discarded. The two fractions were washed in 1.6 ml sterile 1X PBS, centrifuged at 20,000 × *g* and 4°C for 30 min, following which the supernatant was discarded. This wash step was repeated twice, and the pellet was resuspended in 100 μl 1X PBS. Samples for DNA extraction were stored in 1.6 ml Bashing Bead buffer (Zymo research) at −80°C.

Representative samples of the purified fractions collected from the gradients and the original luminal content were stained with Toluidine Blue O (VWR; which specifically binds lignin). They were examined using light microscopy and the size of the stained wood fibers in each fraction was measured using ImageJ.^1^ This step ensured the absence of a size-bias in the recovery of wood fibers from the luminal content.

### 2.3. Preparation of crude enzyme extracts for the assay of cellulase and xylanase activity

Cellulase and xylanase activity in the MG, AHG, and PHG from intact (consisting of the gut tissue and the luminal content) gut compartments were assayed. In order to assay enzymatic contributions from the gut lumen (and not gut tissue), an additional set of assays were carried out for luminal fluid collected from the aforementioned gut compartments. Finally, enzymatic activities in the fiber and fiber-free fractions of the AHG was also assayed. Crude enzyme extracts were prepared and assayed as described by Mikaelyan et al. (2014). The pH of HEPES buffer (Sigma-Aldrich) used for enzyme assays was optimized to represent the native pH in the MG (8.4), AHG (7.2), and PHG (6.8) (Ceja-Navarro et al., 2014). For each replicate, gut compartments from two beetles were placed in clean microcentrifuge tubes containing 200 μl HEPES buffer (of the respective pH) and physically disrupted with sterile micropestles.

Samples from two beetles were utilized for measuring enzyme activities within the gut luminal content, MG, AHG, or PHG (*n* = 4). They were placed in 100 μl sterile HEPES buffer in sterile plastic 100 × 15 mm Petri plates. Gut compartments were split longitudinally, using a sterile razor blade, to disrupt the peritrophic membrane (in the MG). The luminal content was gently mixed with HEPES buffer and transferred to clean 2.0 ml microcentrifuge tubes. Fresh Petri plates were used for every replicate.

Samples were sonicated using a QSONICA Q500 sonicator (QSONICA) at 25% intensity (i.e., set to 125 W), using six cycles of 5 s with a 10-s pause between cycles. Any resultant debris were pelleted by centrifugation at 20,000 × *g*, for 10 min at 4°C. The supernatant, hereafter referred to as the “sonication extract,” was collected in a sterile tube and stored at 4°C before use. The pellet was washed three times with 100 μl protease inhibitor solution (Roche), and resuspended in 100 μl CelLyticB (Sigma-Aldrich) by vortexing it for 15 s to ensure the complete extraction of any potentially cell (wall or membrane) associated enzymes. The final suspension was then placed on ice for 10 min, and briefly centrifuged. The resultant supernatant, hereafter referred to as the “detergent extract,” was collected in a fresh microcentrifuge tube.

For the evaluation of cellulase and xylanase activities, 30 μl of the sonicated or detergent extracts from each sample were incubated with 200 μl of 2.0% [w:v] microcrystalline cellulose (Alfa Aesar) or 0.025% xylan from corn core (TCI), respectively. The reaction mixtures were gently agitated for 1 h at 37°C. Reducing sugar equivalents released during the incubation were colorimetrically estimated at 660 nm on an Ultrospec 7000 (Biochrom) using Mikaelyan et al.’s (2014) modification of the Jue and Lipke (1985) method. Glucose (for cellulase) or xylose (for xylanase) (Tokuda et al., 2018) were used to generate standard curves used to quantify amounts of reducing sugar in the reaction mixtures. One unit of enzyme activity is defined as the amount of enzyme required to release 1 μmol of reducing sugar equivalents from the substrate (cellulose or xylan) per minute per gram of insect.

### 2.4. DNA extraction

Deoxyribonucleic acid was extracted using the Quick-DNA™ Fecal/Soil Microbe Microprep Kit (Zymo research) for the following samples: the MG, AHG, and PHG total luminal content (separated from host tissue), AHG fiber fraction, and AHG fiber-free fraction (prepared using density gradient centrifugation). DNA extractions were done following the manufacturer’s instructions, with three modifications: (1) prior to the bead beating step, microcentrifuge tubes containing the sample and Bashing Bead™ buffer were incubated for 1 h at 65°C with periodic end-to-end inversion to optimize cell lysis; (2) samples were homogenized using a Benchmark Beadblaster 24 homogenizer at 6.0 m/s performed in four cycles of 11 s, with a 30-s rest between cycles; and (3) finally, 600 μl of supernatant was mixed with the Genomic Lysis Buffer instead of the recommended 400 μl. Extracted nucleic acids were stored at −20°C until use.

### 2.5. Estimation of bacterial abundance using qPCR

Bacterial density of luminal content and fiber/fiber-free communities was estimated via qPCR and by using the primers 343F (5′-TACGGGWGGCWGCA-3′) and 784R (5′-GGGTMTCTAATCCBKTT-3′) (Köhler et al., 2012). The final density estimates were normalized to the extracted DNA yields. The reactions were prepared by mixing genomic DNA (10 ng) in a final volume of 20 μl containing 10 μl PowerUp™ SYBR™ Green Master Mix (Applied Biosystems), 1 μl each forward and reverse primer (0.4 μM each), and molecular-grade water. Each reaction consisted of the following steps: activation of Uracil-DNA glycosylases in the master mix for 2 min at 50°C, a 2-min DNA denaturation step at 95°C and finally, 39 cycles of 5 s at 95°C, 15 s at 55°C, and 10 s at 72°C. 16S rRNA genes in the samples were quantified using standard curves generated using known quantities of *E. coli* strain DH5α 16S rRNA genes that were amplified using primers 27F and 1492R (Lane, 1991). The total number of 16S rRNA gene fragments was calculated using a known number of *E. coli* 16S rRNA gene amplicons as a standard and the total number of 16S rRNA genes in each reaction were calculated using the Bio-Rad CFX Maestro software (version 2.0, Bio-Rad).

### 2.6. Amplicon sequencing of the 16S rRNA gene

Deoxyribonucleic acid extracted from the luminal content and Percoll fraction samples was used to generate a total of 9 MiSeq amplicon libraries that targeted the hypervariable V3-V4 region of the 16S rRNA gene. Forward and reverse primers included unique 8-base barcodes at the 5′ end. PCR was performed with 50 μl reactions, which contained ∼10 ng of template DNA, 0.4 μM forward primer (S-D-Bact-0341-b-S-17; 5′-CCTACGGGNGGCWGCAG-3′) (Klindworth et al., 2012), 0.4 μM reverse primer (S-D-Bact-0785-a-A-21; 5′-GACTACHVGGGTATCTAATCC-3; Klindworth et al., 2012), and 25 μl Taq polymerase master mix red (PCRBIO). The following cycling conditions were used: 30 s of denaturation at 95°C, 25 cycles of 20 s at 95°C, 20 s at 58°C, 30 s at 72°C, and a final elongation step at 72°C for 3 min. Amplicons were cleaned using the DNA Clean and Concentrator-5 Kit (Zymo research) following the manufacturer’s instructions. Purified amplicons were quantified and commercially sequenced at Novogene (Beijing, China) using the Illumina MiSeq platform.

### 2.7. Sequence quality control and processing

Sequence reads obtained from the samples were primarily processed using commands implemented in the program suite Mothur (v1.44.1; Schloss et al., 2009), unless specified otherwise. Contigs produced from the paired fastq files were subjected to stringent quality control and only reads with a minimum length of 200 bases, an average quality score greater than 25, no ambiguities, and a maximum homopolymer length of ten bases were retained. Chimeric sequences were removed and quality-filtered sequences were clustered, using a 97% similarity threshold using vsearch [v2.14.2; (Rognes et al., 2016)], to define *de novo* operational taxonomic units (OTUs). Representative sequences from each OTU were aligned and classified taxonomically with the Ribosomal Database Project (RDP) Naïve Bayesian Classifier (Wang et al., 2007) implemented in Mothur using the taxonomic framework of the SILVA non-redundant database (v.138) (Quast et al., 2013; Yilmaz et al., 2014). A phylogenetic tree of all OTU representatives was constructed using FastTree [v2.1.3; (Price et al., 2010)] that was used for the calculation of UniFrac (Lozupone and Knight, 2005) beta-diversity distances between samples in Mothur.

### 2.8. Microbiome data analysis

The bacterial diversity in the samples was analyzed using R (version 4.0.4; R Core Team, 2021). The OTU table and taxonomy file from the sequence processing pipeline were combined into a *phyloseq* (Mcmurdie and Holmes, 2013) “object” to aid in data management. Rare taxa that represented fewer than 10 reads were removed using the *filter_taxa* function. Sequences that could not be classified even at the phylum level, or those classified as eukaryotic organelles, were eliminated from downstream analysis using the *subset_taxa* function. Samples were rarefied to the lowest number of quality-filtered reads obtained from an individual sample (4,085 sequences) using the *rarefy_even_depth* function with the rngseed value set to 81. Weighted UniFrac (Lozupone and Knight, 2005) distances were calculated using the *unifrac.weighted* function in Mothur (v1.44.1; Schloss et al., 2009). Weighted UniFrac distances were used to test for differences in community structure using permutational multivariate ANOVA and differences were visualized using non-metric multidimensional scaling; both analyses were performed using the package *vegan* (Oksanen et al., 2017) using the functions *adonis2* and *metaMDS*, respectively. Ordinations were plotted using the *ordiplot* and *ordihull* functions in *vegan*. Finally, differential abundance analysis was performed with the R package *DESEQ2* (Love et al., 2014) to better understand the differences in community structure between the different fractions obtained from the Percoll gradient. Results from the *DESEQ2* analysis were visualized by generating a heatmap using the *heatmap.2* function in the R package *gplots* (Warnes et al., 2016).

### 2.9. Phylogenetic relationships within fiber-enriched bacterial taxa

In order to identify the phylogenetic positions of the bacterial phylotypes preferentially enriched on wood fibers in *O. disjunctus*, we conducted phylogenetic analyses for two genera *Lactococcus* and *Turicibacter*. Considering their substantial presence on wood fibers, we also comprehensively investigated the evolutionary context of genus-level Christensenellaceae R-7 group and *Treponema* to expand further our understanding of their ecological distribution. In order to overcome the severe limitations on phylogenetic resolution when using short (∼0.4 kb) 16S rRNA reads, we amplified the (∼4 kb) 16S-23S region of the rRNA operon using universal primers following a previously described approach (Martijn et al., 2019). Briefly, we used the forward (A519F 5′-CAGCMGCCGCGGTAA-3′) and reverse (U2428R 5′-CCRAMCTGTCTCACGACG-3′) primers (with unique 8-base barcodes attached to the 5′ end) to amplify the 16S-23S region using the following PCR cycling conditions: denaturation at 98°C for 30 sec followed by 30 cycles of 98°C for 10 s, annealing at 64°C for 30 s, extension for 3 min and 30 s at 72°C, and a final extension step at 72°C for 10 min. Amplicons were purified using the Select-a-Size DNA Clean and Concentrator MagBead Kit (Zymo) following the manufacturer’s instructions with one modification–samples were mixed at a [10:4] ratio with the MagBead buffer. Amplicons obtained from both *O. disjunctus* fiber and luminal fluid samples were then sequenced using the PacBio Sequel I system at the NCSU Genomic Sciences Laboratory. Circular consensus sequencing (CCS) reads were generated downstream from the raw reads using the CCS function (v 6.4.0) in the PacBio *bioconda* tool.^2^ Next, the 16S rRNA region of the amplicon was extracted using barrnap (version 0.9).^3^ Sequences were aligned using Mothur against the “silva.gold.align” reference database, imported into the full SILVA v 138.1 reference database in ARB (Ludwig et al., 2004), and placed phylogenetically into the SILVA “Tree of Life” using ARB’s quick-add by parsimony tool.

For all four taxa of interest (*Lactococcus, Turicibacter, Treponema*, and Christensenellaceae R-7), ingroup, outgroup, and root sequences were selected to appropriately represent different levels of the phylogenetic hierarchy. Special attention was given to the selection of standard (American Type Culture Collection, ATCC) strains in the ingroup and outgroups to provide phylogenetic context. Potentially chimeric sequences with pintail scores < 50 were excluded from the analysis, and sequence redundancy was reduced by OTU selection at 98% identity using Vsearch (Rognes et al., 2016). The alignments of sequences obtained in this study and the reference sequences from the Silva database were exported from ARB in fasta format for phylogenetic analysis with IQTREE2 (Minh et al., 2020). Tree calculations included model selection (Kalyaanamoorthy et al., 2017) and branch support using 1,000 Ultrafast Bootstrap replicates (Hoang et al., 2018). Finally, clades with insect-associated sequences were identified within the constructed phylogenies and assigned labels in ARB that would inform their host distribution. This updated version of the database was exported and used as a reference for taxonomic reassignment of short read 16S rRNA amplicon sequences (obtained as described in the previous section) using Mothur (v1.44.1; Schloss et al., 2009). The constructed trees were imported into ARB, and the environmental origin in the sequence metadata of the Silva reference database was used to identify clades predominantly or exclusively associated with insects. This information was used to increase the taxonomic resolution of the short-read Illumina sequences by reclassifying them with Mothur as described in the section above (v1.44.1; Schloss et al., 2009).

### 2.10. Scanning electron microscopy

Wood fibers colonized by bacteria were collected from the AHG of an adult *O. disjunctus*. The gut contents were allowed to sediment via gravitation and the supernatant was removed. The wood fibers were resuspended in 2.5% glutaraldehyde in 0.1 M sodium cacodylate buffer, pH 7.4 (Electron Microscopy Sciences), for 1 h at room temperature and stored at 4°C. An aliquot was transferred to a 1.5 ml microcentrifuge tube and washed three time with 0.15 M sodium phosphate buffer, pH 7.4. They were post-fixed in 1% osmium tetroxide in 0.15 M sodium phosphate buffer, pH 7.4 for 1 h. Samples were washed in deionized water three times, transferred into type-A microporous specimen capsules of 120–200 μm pore size (Ted Pella) and were gradually dehydrated with ethanol (30, 50, 75, 100, and 100%). The capsules were transferred to a Samdri-795 critical point dryer and dried using carbon dioxide as the transitional solvent (Tousimis Research Corporation). Contents of the capsule were gently transferred onto double-sided carbon adhesives placed on 13-mm aluminum stubs. They were then sputter coated with 11 nm of gold-palladium alloy (60 Au:40 Pd, Cressington Sputter Coater 208HR, model 8,000–220). Specimens were examined and images taken using a Zeiss Supra 25 FESEM operating at 5 kV, using the InLens detector, ∼5-mm working distance, and 30-μm aperture (Carl Zeiss SMT Inc.).

## 3. Results

### 3.1. Bacterial density along the gut axis

**FIGURE 1:**
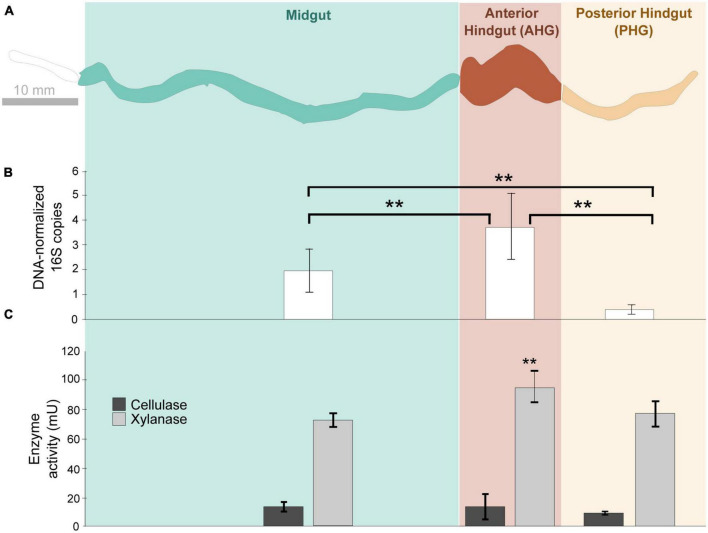
Schematic diagram **(A)** of the gut compartments of *Odontotaenius disjunctus* analyzed for bacterial density **(B)** and enzyme (cellulase and xylanase) activity **(C)**. Bacterial density in the compartments is represented as normalized counts of qPCR-amplified 16S rRNA genes. One unit of enzyme activity is defined as 1 μmol of sugar equivalent released per minute, per gram of insect. **marks significant differences (*P* ≤ 0.001).

Bacterial density, estimated via qPCR of 16S rRNA genes, was observed to be significantly different among luminal content from the midgut (MG), anterior hindgut (AHG), and posterior hindgut (PHG) (ANOVA: *F* = 105.6; *p* < 0.001; Figure 1B). Of the three gut compartments, the *O. disjunctus* AHG had the highest density of 16S rRNA genes at 3.78 × 10^9^ ± 1.49 × 10^9^ genes^–1^ DNA extraction (mean ± standard deviation; Figure 1B), followed by the MG (1.98 × 10^9^ ± 9.55 × 10^8^ genes^–1^ DNA extraction) and the PHG (3.99 × 10^8^ ± 2.38 × 10^8^ genes^–1^ DNA extraction) compartments.

### 3.2. Cellulase and xylanase activity along the gut axis of *Odontotaenius disjunctus*

Lumen cellulase activity was not significantly different among the MG (14.35 ± 3.25 mU g^–1^ beetle), AHG (14.41 ± 8.59 mU g^–1^ beetle), and PHG (10.03 ± 1.18 mU g^–1^ beetle; ANOVA: *F* = 2.007, *p* = 0.197; Figure 1C). However, lumen xylanase activity was significantly different among the MG, AHG, and PHG (ANOVA: *F* = 4.915, *p* = 0.045, Figure 1C). Pairwise comparisons of the luminal fluid samples revealed that the xylanase activity in the AHG lumen (95.47 ± 10.71 mU g^–1^ beetle) was significantly higher than that estimated both in the MG (73.15 ± 4.74 mU g^–1^ beetle; *p* = 0.0215) and PHG (78.03 ± 8.66 mU g^–1^ beetle; *p* = 0.0215; Figure 1C). When the activities in the hindgut compartments were combined (AHG + PHG), the hindgut lumen (24.44 ± 9.78 mU g^–1^ beetle for cellulase and 173.49 ± 7.25 mU g^–1^ beetle for xylanase; Figure 1) was significantly higher than the MG compartment (14.36 ± 3.25 mU g^–1^ beetle for cellulase (*p* = 0.048) and 73.15 ± 4.73 mU g^–1^ beetle for xylanase (*p* < 0.001); Figure 1C).

Strikingly, both enzymatic activities observed in the intact MG compartment, including the gut tissue (69.4 ± 38.28 and 333.67 ± 10.80 mU g^–1^ beetle, respectively; for details on intact activity Supplementary Table 1), was substantially higher than in the MG lumen alone (cellulase: 14.36 ± 3.25 mU g^–1^ beetle; xylanase: 73.15 ± 4.73 mU g^–1^ beetle; Figure 1C). The MG lumen only retained 20.6% of the total cellulase and 21% of total xylanase activity in the intact compartment (See Supplementary Table 2 for proportions of activity retained by the lumen). In contrast, there were no significant differences observed between activities assayed from the lumen and intact compartments in the hindgut. For the AHG, luminal cellulase (14.42 ± 8.59 mU g-1 beetle; Figure 1C) and xylanase activity (95.47 ± 10.71 mU g-1 beetle) did not differ significantly from intact compartments (cellulase: 9.89 ± 4.46 mU g-1 beetle; ANOVA: *F* = 0.69, *p* = 0.413 and xylanase: 109.26 ± 13.20 mU g-1 beetle; ANOVA: *F* = 2.63, *p* = 0.156; Supplementary Table 1). Similarly, xylanase activity in the PHG lumen (78.03 ± 8.66 mU g^–1^ beetle; Figure 1C) was not significantly different from that observed in the intact PHG (68.14 ± 9.81 mU g^–1^ beetle; ANOVA: *F* = 0.215, *p* = 0.659; Supplementary Table 1). Taken together, these observations suggest that while a significant amount of enzymatic activity in the MG is associated with the epithelium, most of the activity in hindgut compartments are associated with the lumen.

### 3.3. Imaging of wood particles from the anterior hindgut

**FIGURE 2:**
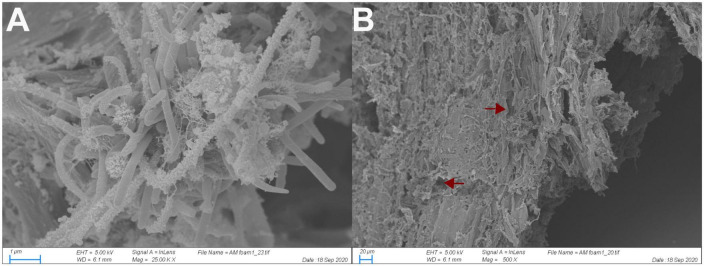
Scanning electron micrograph of bacterial cells adhering to wood fibers in the anterior hindgut of *O. disjunctus* **(A)** and rough degradation of the wood fiber surface **(B)**. Arrows point to areas with likely fungal degradation.

The scanning electron micrographs revealed that wood particles extracted from the luminal content of the *O. disjunctus* AHG are densely colonized by biofilms composed of various rod-shaped and filamentous cells (Figure 2A). Many cells possessed a number of surface protuberances that resembled multi-enzyme complexes called cellulosomes (Lamed et al., 1987), or analogous adaptations commonly found in anaerobic bacteria associated with plant material. Although no fungal mycelia could be definitively identified in our micrographs, the holes observed in the wood fibers (Figure 2B) are suggestive of fungal degradation of wood prior to ingestion.

### 3.4. Enrichment of wood particles from the anterior hindgut using discontinuous density gradient centrifugation

**FIGURE 3:**
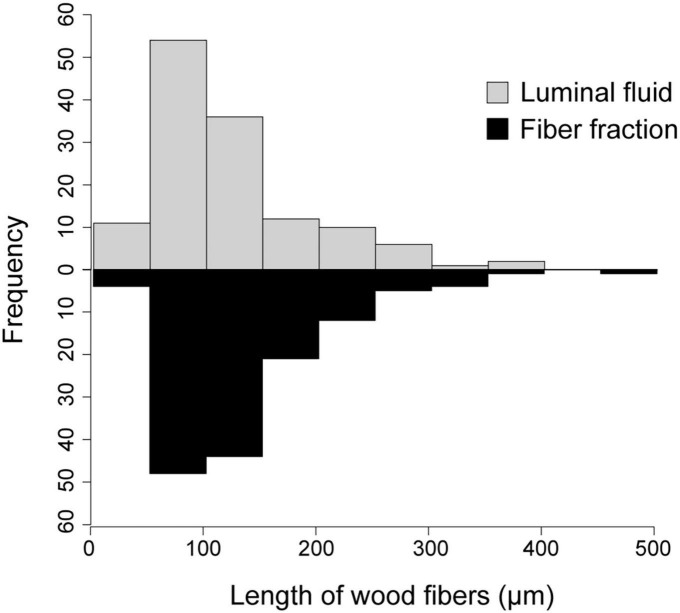
Histogram of wood fiber lengths in the AHG lumen of *O. disjunctus* and in the fiber fraction obtained from Percoll density-gradient centrifugation of luminal fluid.

Upon density gradient centrifugation, the luminal contents of the anterior hindgut (AHG) of *O. disjunctus* separated into two distinct turbid bands. One of them was a lightly colored one banding at a density of around 1.04 g/ml (“the fiber-free fraction”; See Supplementary Figure 1 for separation of density marker beads in the density gradient) and the second was a much darker, denser band with a density of 1.13 g/ml (“the fiber fraction”). Light microscopic examination of the two purified fractions, stained with Toluidine Blue O, confirmed that the heavier fiber fraction exclusively contained lignin-rich wood particles (data not shown). In contrast, the fiber-free fraction contained planktonic bacterial cells with only a few tiny or no wood particles. Furthermore, a comparison of the size distribution of wood particles in the AHG luminal content (124.6 ± 8.14 μm) of *O. disjunctus* and the fiber fraction (133.5 ± 6.18 μm) demonstrated no significant difference (ANOVA: *F* = 0.77; *p* = 0.37; Figure 3), thus indicating that no selective loss of wood particles of particular lengths occurred as a result of the fiber enrichment protocol.

### 3.5. Distribution of cellulase activity and 16S rRNA genes in fractionated luminal content

**FIGURE 4:**
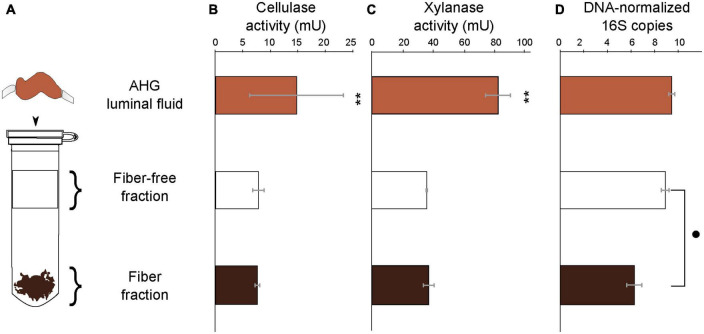
Schematic representation **(A)** of the Percoll fractionation of the luminal content in the AHG of *O. disjunctus* into the Fiber-free and Fiber fractions, cellulase activity **(B)**, xylanase activity **(C)**, and bacterial density **(D)**. Bacterial density in the compartments is represented as normalized counts of qPCR-amplified 16S rRNA genes. One unit of enzyme activity is defined as 1 μmol of sugar equivalent released per minute, per gram of insect. **and mark significant differences at the *P* ≤ 0.001 and *P* ≤ 0.05 levels.

Cellulase activity did not differ significantly (*t* = −0.81 *p* = 0.44) between the fiber (7.53 ± 0.38 mU g^–1^ beetle) and fiber-free fractions (7.76 ± 0.31 mU g^–1^ beetle; Figure 4 and Supplementary Table 3) obtained from the anterior hindgut (AHG) lumen of *O. disjunctus*. Xylanase activity similarly did not differ significantly (*t* = 0.52: *p* = 0.69) between the fiber (43.13 ± 4.67 mU g^–1^ beetle) and fiber-free (41.90 ± 0.46 mU g^–1^ beetle) fractions from the AHG lumen. When combined, the cellulase activity (15.28 ± 0.56 mU g^–1^ beetle) and xylanase activity (70.85 ± 3.68 mU g^–1^ beetle) in the fiber and fiber-free fractions was not significantly different (cellulase: *t* = 0.02, *p* = 0.92 and xylanase: *t* = −1.008, *p* = 0.14) from that of the AHG luminal content (cellulase: 14.42 ± 8.59 mU g^–1^ beetle and xylanase: 95.46 ± 0.46 mU g^–1^ beetle). These results suggest that almost all of the enzyme activity in the luminal fluid was recovered in the two Percoll fractions. The fiber fraction of the *O. disjunctus* AHG (3.65 × 10^6^ ± 3.06 × 10^6^ genes^–1^ DNA extraction) had significantly fewer normalized 16S rRNA genes than did the fiber-free fraction (1.06 × 10^9^ ± 7.31 × 10^8^ genes^–1^ DNA extraction; Wilcoxon: *p* = 0.02; Figure 4 and Supplementary Table 3).

### 3.6. Microbiome analysis

**FIGURE 5:**
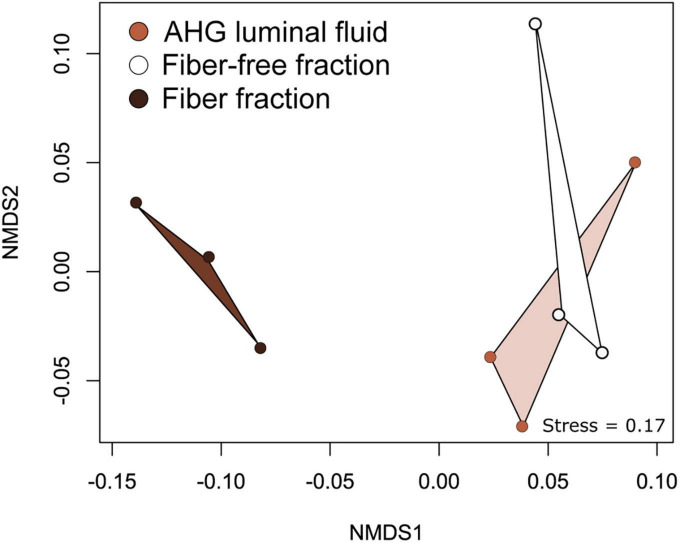
Non -metric Multidimensional Scaling (NMDS) analysis of weighted UniFrac distances between bacterial communities associated with AHG luminal content, the fiber and fiber-free fractions obtained from Percoll density gradient centrifugation.

We sequenced the amplified V3-V4 region of the 16S rRNA genes from luminal content, the fiber fraction, and the fiber-free fraction collected from *O. disjunctus*. Following data processing in Mothur, samples were rarefied such that there were 4,085 sequences per sample. Using the SILVA (v138) (Quast et al., 2013; Yilmaz et al., 2014) non-redundant database, we were able to classify 69% of sequences to the genus level. We created 3,285 OTUs that could be assigned to 495 genera in the curated dataset, which was used for downstream analyses (See Supplementary Table 5 for the distribution and abundance of OTUs in the full dataset). Ordination analysis based on weighted UniFrac distances showed that the community structure was significantly affected by the sample type (PERMANOVA: *F* = 4.06, *R*^2^ = 0.474, *p* = 0.0015; Figure 5). In ordination space, the fiber-associated community samples were clearly separated from the samples of fiber-free and luminal content communities, which clustered together (Figure 5).

**FIGURE 6:**
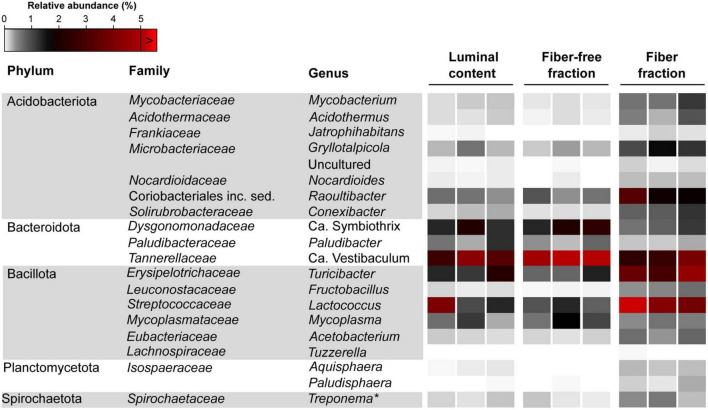
Relative abundance of bacterial genera differentially abundant between the fiber and fiber-free community across all samples. *Termite *Treponema* cluster.

*DESEQ2* analysis identified the top 20 genera which were differentially abundant between the fiber-associated and fiber-free communities (Figure 6; See Supplementary Table 4 for detailed statistics and Supplementary Table 5 for the relative abundance of all bacterial taxa across samples). Collectively, these taxa represented 23.07 ± 3.58%, 12.83 ± 1.80%, and 12.32 ± 0.29% of the fiber-associated, fiber-free, and luminal content communities, respectively (Supplementary Table 3). Of these 20 genera, 16 were significantly more abundant in the fiber-associated community than in the fiber-free community, while the remaining four were significantly more abundant in the latter (Supplementary Table 4.) Of the 16 genera that were enriched in the fiber-associated community, only 4, *Lactococcus* (*Streptococcaceae*), *Raoultibacter* (*Coriobacteriales* inc. sed.), *Turicibacter* (*Erysipelotrichaceae*), and *Gryllotalpicola* (*Microbacteriaceae*) represented at least 1% total relative abundance of the fiber-associated community.

Among Bacillota, *Lactococcus* (*Streptococcaceae*; 6.57 ± 3.60%; Wald = 4.61, *p* < 0.001) and *Turicibacter* (*Erysipelotrichaceae*; 3.66 ± 0.71%; Wald = 4.73, *p* < 0.001) were significantly enriched in the fiber-associated community compared to the free-living, fiber-free, community. Combined, these two genera represent 10.24 ± 4.32% of the fiber community, whereas they only represent 1.92 ± 0.58% of the fiber-free community. The relative abundance of the genus *Lactococcus* differed more drastically between the fiber and fiber-free communities. *Lactococcus* members in the fiber-associated community were observed to be ∼700% more abundant compared to the fiber-free community, whereas members of *Turicibacter* showed only a ∼380% increase. The other two genus level groups, significantly enriched in the fiber fraction belonged to the phylum Acidobacteriota: *Gryllotalpicola* (*Microbacteriaceae*; 1.2 ± 0.26; Wald = 3.95, *p* = 0.001) and *Raoultibacter* (*Coriobacteriales*; 2.34 ± 0.65; Wald = 3.95, *p* = 0.001), representing a combined 3.54 ± 0.91% of the fiber-associated community. Compared to the fiber-free community, members of *Gryllotalpicola* and *Raoultibacter* were ∼400% more abundant in the fiber-associated community.

The remaining four genus-level groups that were more selectively enriched in the fiber-free community, *Candidatus* Symbiothrix (*Dysgonomonadaceae*; 1.98 ± 0.48%; Wald = 3.90, *p* = 0.001), *Candidatus* Vestibaculum (*Tannerellaceae*; 5.02 ± 0.85%; Wald = 3.52, *p* = 0.004), and *Mycoplasma* (*Mycoplasmataceae*; 1.06 ± 0.47%; Wald = 2.99, *p* = 0.002) approximately represented a combined 8% of the fiber-free community while only 4.3% of the fiber-associated community. Additionally, *Paludibacter* (*Paludibacteraceae*; 0.44 ± 0.19%; Wald = 2.83, *p* < 0.001) was enriched in the fiber-free community–roughly double the relative abundance of *Paludibacter* in the fiber-associated community (0.24 ± 0.05%).

Other genus-level groups exhibited notable abundance not only in the fiber fraction but also in the luminal fluid and fiber-free fraction, thereby suggesting that they may not be specifically associated with wood fibers. Among these groups, Subgroup 18 (Acidobacteriota) constituted 6.31% of the fiber fraction, 15.89% of the luminal fluid, and 14.62% of the fiber-free fraction (Supplementary Table 5). Similarly, the ubiquitous presence of the Christensenellaceae R-7 group was observed, representing approximately 8-9% of the luminal fluid, fiber-free fraction, and fiber fraction (Supplementary Table 5). Another prominent group, the genus *Treponema*, accounted for 6.00% of the fiber fraction, and approximately 6.57% and 8.9% of the luminal fluid and fiber-free fraction, respectively (Supplementary Table 5).

### 3.7. Phylogenetic analysis for groups of interest

**FIGURE 7:**
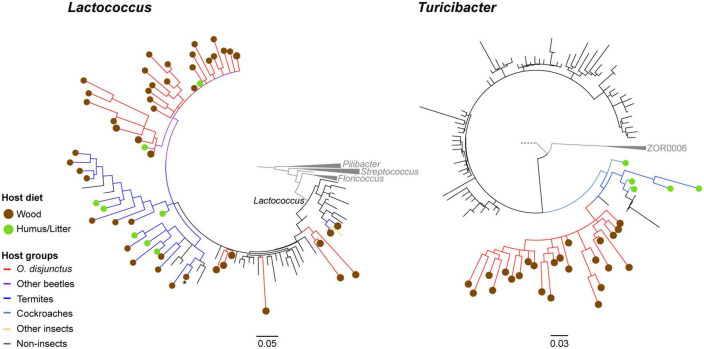
Consensus trees based on partial 16S rRNA genes for 1,000 ultrafastbootstrap replicates of *Lactococcus* (calculated with 129 sequences and 1,570 columns) and *Turicibacter* (calculated with 681 sequences and 1,590 columns). Nodes showing less than 60% support have been collapsed into multifurcations. Edges and circles are, respectively colored by diet and taxonomy of the hosts, from which the sequences were obtained.

Phylogenetic analysis of longer PacBio-sequenced regions of the 16S rRNA genes from *Lactococcus* and *Turicibacter* revealed interesting patterns of host-specificity. For both phylogenies we largely recovered topologies similar to what was seen in the tree of life according to SILVA (v138) (Quast et al., 2013; Yilmaz et al., 2014). The phylogenies of groups of interest revealed that members from the hindgut of *O. disjunctus* are closely related to representatives found in other scarab beetles, termites, and cockroaches (Figure 7).

Phylogenetic placement of OTU centroids identified as *Lactococcus* were observed to be closely related to both termite- and beetle-associated sequences. We also recovered a clade of sequences identified as the genus *Lactovum*, associated almost exclusively with termites, nested within the insect-associated *Lactococcus* clade. One of the clades contained a sequence from a cultured isolate of *Lactococcus* isolated from the gallery of an unidentified passalid beetle from Costa Rica (Vargas-Asensio et al., 2014). Within that same radiation (Figure 7) is a *Lactococcus* representative isolated from the hindgut wall of cane beetle larvae, *Dermolepida albohirtum* (Pittman et al., 2008) and a larva of the Japanese rhinoceros beetle, *Trypoxylus dichotomus*. Additionally, *Lactococcus* sequences from the *O. disjunctus* gut clustered closely with representative sequences from the guts of multiple termite groups including *Coptotermes*, *Reticulitermes*, *Nasutitermes*, and *Amitermes*. Similar patterns were observed for the *Turicibacter* phylotypes obtained from the *O. disjunctus* gut that were closely related to members sequenced from both two lignocellulose-feeding beetles *Pachnoda ephippiata* (Coleoptera: *Scarabaeidae*) and *Protaetia aurichalcea* (Coleoptera: *Scarabaeidae*; Figure 7). Based on these results we established that these were insect-associated clades within *Lactococcus* and *Turicibacter*. Using these new phylogenies as taxonomic frameworks, we were able to classify short read 16S rRNA sequences from this study, and confirmed that the short reads classified to these taxa were exclusively members of these insect-specific clades.

Phylogenetic examination of the centroid sequences within the genus *Treponema* showed them to be affiliated with *Treponema* I (*sensu* Mikaelyan et al., 2015; Supplementary Figure 2), a genus-level taxon traditionally believed to be restricted in their distribution to termites (Mikaelyan et al., 2015). The classification of our short reads to *Treponema* (*sensu stricto*) indicate a potential misassignment, possibly due to a paucity of comprehensive reference sequences from *Odontotaenius disjunctus* in public databases.

Our analysis of the Christensenellaceae R-7 group in *O. disjunctus* unveiled a remarkable diversity among the centroid sequences, distributed across the phylogenetic tree in multiple distinct clades (Supplementary Figure 3). Nevertheless, numerous centroids exhibited close proximity to sequences derived from wood- or humus/litter-specializing cockroaches and termites, hinting at potential ecological associations and adaptation within these wood-feeding or detritivorous insect groups (Clades Ai and Bi; Supplementary Figure 3).

## 4. Discussion

Our study of *Odontotaenius disjunctus* reveals three key insights into symbiotic wood digestion in passalid beetles. First, the hindgut of *O. disjunctus* is the primary site of cellulose and hemicellulose (xylan) degradation in the gut (Figure 1 and Supplementary Table 2). These results thereby revise the current hypothesis (Ceja-Navarro et al., 2019) about the midgut (MG) being the primary site of bacterial depolymerization of lignocellulose. Second, the role of the fiber-associated microbiome in the anterior hindgut appears to be pivotal, accounting for a significant proportion of the luminal cellulase and xylanase activity in the anterior hindgut. This contribution is further emphasized as it also accounts for as high as 19% of the total assayed cellulase or xylanase activity in the gut (Figures 1, 4 and Supplementary Tables 2, 3). Third, the consistent association (Figure 5) of specific insect-restricted bacterial groups (Figures 2, 6) with the wood particles isolated from the AHG clearly indicates that the particles are colonized by a specialized fiber-associated microbiome. We further demonstrate that this fiber-associated microbiome contributes substantially to the cellulase and xylanase activity in the hindgut, and forms a key symbiotic component in the beetle’s lignocellulose digestion machinery. The central role of the fiber-associated microbiome in the anterior hindgut of *O. disjunctus* points to a remarkable convergence in the evolutionary trajectories that have independently evolved in at least two wood-feeding insect groups, namely, the termites and passalid beetles.

### 4.1. The compartmentation of xylanase and cellulase activity in the gut of *O. disjunctus*

The digestive division of labor between the midgut and the hindgut of *O. disjunctus* broadly reflects the model of “dual cellulose digestion” observed in termites, and suggests that the sequential action of enzymes in the midgut and hindgut are responsible for the symbiotic digestion of lignocellulose. However, there appear to be marked differences displayed by the two insect groups in the precise pattern of distribution of cellulase and xylanase activities within and between the two gut compartments. The distribution of cellulase activity in *O. disjunctus* intact gut compartments suggests that the hindgut retains only around 53 and 21% of xylanase and cellulase activity compared to the midgut (Figure 1 and Supplementary Table 1). In contrast, the hindgut of wood-feeding higher termite *N. takasagoensis* retains about 5,933% for xylanase (Tokuda et al., 2018) and ∼59% for cellulase (Tokuda and Watanabe, 2007).

At first glance, this stark contrast between *O. disjunctus* and termites might indicate that, lignocellulose digestion in *O. disjunctus* is skewed more to favor endogenous enzymes in the midgut than microbial enzymes in the hindgut. However, our analysis of the sub-compartmentalization of activities within the midgut of *O. disjunctus* suggest a potential overestimation of enzyme activity available for the digestion for wood fibers in the lumen. The majority of enzyme (78% of xylanase and 80% of cellulase; Supplementary Table 2) activity in the midgut is retained in the tissue, likely not contributing to luminal digestion of wood particles in the endoperitrophic space. On the contrary, these data suggest that most of the midgut-associated activity appears to be involved in the terminal digestion of oligosaccharides and disaccharides at the epithelium. Interestingly, the opposite appears to be true for wood-feeding termite *Nasutitermes walkeri* (related to the *N. takasagoensis* mentioned earlier), where it is the midgut lumen that retains the bulk of the cellulase activity (Hogan et al., 1988). Therefore, in order to more accurately compare digestive capabilities between the midgut and hindgut, it is more appropriate to compare the values for the lumen of the hindgut and midgut of *O. disjunctus*, which eliminates potential confounding factors related to enzyme distribution in the tissue. On applying this correction, the retention of 237% of xylanase activity and 170% of cellulase activity (compared to the midgut lumen; Figure 1) by the hindgut lumen suggests a stronger role for the hindgut as the site of lignocellulose digestion than previously inferred (Ceja-Navarro et al., 2019). However, since we limited our analysis to xylanase and cellulase activities, we cannot speak directly about the relative role of the hindgut in lignin depolymerization, which may still be favored in the more oxygenated midgut (Ceja-Navarro et al., 2019).

The differences we observed in sub-compartmentalization of enzyme activity of the midgut in termites and *O. disjunctus* provides insights into potential differences in digestive strategies between the two wood-feeding groups. However, further investigation is needed into how different components of lignocellulose might be degraded through gut passage, including comprehensive assessments of the type of wood consumed, enzymatic activities, rate of feeding, and gut retention time. Continued research in this area will contribute to a more nuanced understanding of the intricate factors influencing digestive efficiency in these organisms.

Indeed, our observation that the anterior hindgut compartment is home to the densest bacterial community of all three gut compartments (anterior hindgut, posterior hindgut, and midgut; Figure 1) supports the previously described role (Ceja-Navarro et al., 2019) of the anterior hindgut as the primary fermentation chamber in *O. disjunctus*. This also highlights how the AHG in *O. disjunctus* is functionally analogous to the anterior P3 compartment of the hindgut in higher termites (Brune and Dietrich, 2015). The posterior hindgut (PHG) contributed almost as much as the AHG in terms of cellulase and xylanase activities, however, it was several-fold lower in bacterial density (Figure 1). Electron micrographic evidence from *O. disjunctus* suggests that the less diverse microbial community at the PHG epithelium was characterized not only by fewer bacteria than the AHG, but by “scattered clumps” of filamentous yeast (Nardi et al., 2006). Furthermore, cultivation-based evidence suggests that the posterior hindgut is home to many species of cellobiose- and xylose-fermenting yeast, which have been suggested to play roles in the terminal digestion of polysaccharides in the PHG (Suh et al., 2003; Urbina et al., 2013). It is therefore plausible that, given the low abundance bacteria in the PHG, the activities we assayed include contributions from still poorly uncharacterized yeasts in the posterior hindgut.

### 4.2. The role of the fiber-associated microbiome in lignocellulose digestion

The difference in bacterial density between the anterior and posterior hindgut, despite similar enzyme activity, suggested that exploring the composition and enzymatic contributions of the fiber-associated community in the anterior hindgut is necessary to understand the bacterial contribution to overall wood digestion in *O. disjunctus*. The presence of a cellulolytic and xylanolytic fiber-associated microbiome in the AHG of *O. disjunctus* highlights the importance of wood particles as a microhabitat for fiber-digesting bacteria. The retention of equivalent amounts of enzyme activity in the fiber and fiber-free fractions (Figures 4B, C) strongly suggests that lignocellulose digestion in the AHG is highly compartmentalized, and that bacterial members in both the fiber-associated and planktonic communities contribute to xylan and cellulose digestion. Notably, although similar proportions of enzyme activity were associated with the two community fractions, the bacterial density in the fiber-associated community is two orders of magnitude lower than the planktonic community (Figure 4D), which suggests the importance of this microhabitat in facilitating the colonization of a more ecologically specialized microbiome.

While the colonization of plant material by bacterial communities is to be expected for most environments characterized by anaerobic lignocellulose digestion (Brulc et al., 2009; Mikaelyan et al., 2014; Ransom-Jones et al., 2017; Tokuda et al., 2018), the observation of a fiber-associated community (Figure 2) of specific composition (Figures 5, 6) in *O. disjunctus* strongly suggests a specialized role for these bacterial colonizers. Such reliance on a fiber-associated microbiome for wood digestion presents a scenario that is remarkably similar to symbiotic digestion in wood-feeding higher termites (Mikaelyan et al., 2014; Tokuda et al., 2018). However, there are stark compositional differences between the fiber-associated microbiomes of termites and passalid beetles that are reflected already at the phylum level. While the wood particles in *Nasutitermes* spp. are colonized by a relatively simple microbiome–primarily made up of Fibrobacterota and *Treponema* I (Mikaelyan et al., 2014; Tokuda et al., 2018)–those in *O. disjunctus* are far more diverse, dominated instead by Acidobacteriota, Actinomycetota, Bacteroidota, and Bacillota (Figure 6 and Supplementary Table 4). Intriguingly, the clustering of full-length sequences from *O. disjunctus* within the *Treponema* I group (sensu Mikaelyan et al., 2015; Supplementary Figure 2) indicates that it is more widely distributed among insects that feed on wood and other forms of lignocellulose, challenging the previous notion of their exclusivity to termites (Mikaelyan et al., 2015). Further research could comprehensively elucidate the diversity of *Treponema* I across passalid species, and if it plays a comparable functional role in *O. disjunctus* as it does in termites. Regardless, the fundamental differences in fiber-associated microbiomes suggest that, despite having evolved similar digestive strategies, these unrelated wood-feeding insect groups have recruited different lineages of fiber-digesting symbionts.

The consistent association of *Lactococcus* and *Turicibacter* with fiber fractions from *O. disjunctus* AHG suggests that these members have specialized roles in fiber digestion. Although both genera are widely distributed in intestinal environments, the placement of phylotypes from *O. disjunctus* within insect-specific clades (Figure 7) could reflect different degrees of ecological specialization to the guts of lignocellulose-feeding insects. The almost exclusive association of these insect-specific clades with lignocellulose-feeding hosts, specifically passalid beetles, scarab beetles and termites, further supports this hypothesis. However, the strongest evidence so far of the putative roles of these phylotypes in fiber digestion comes from published genomes and metagenomes. In a previous study, a metagenome-associated genome (MAG; g71c2) encoding a range of glycosyl hydrolases (GH1, GH5, GH8, GH10, and GH43) was binned to the genus *Lactococcus*, identifying it as a key potential contributor to AHG lignocellulose digestion (Ceja-Navarro et al., 2019). The enrichment of *Lactococcus* members in the fiber-associated community of the AHG (Figure 6) provides further evidence in support of their role in digestion. *Lactococcus* sequences from the fiber fraction were placed in the close phylogenetic neighborhood (Figure 7) of *Lactococcus lactis*. INBio_4514B from the gallery of a passalid beetle (Vargas-Asensio et al., 2014) and *Lactococcus lactis* Da-18 from the hindgut of a more distantly related scarab beetle *Dermolepida albohirtum* (Pittman et al., 2008). This finding indicates that this particular clade of lactococci might be exclusively associated with scarab beetles. Although *Lactococcus* does not appear to play a major role in the microbial ecology of the termite gut (Dietrich et al., 2014; Mikaelyan et al., 2015), its enrichment in the crop of the wood-feeding cockroach *Panesthia angustipennis* (Bauer et al., 2015; Lampert et al., 2019) indicates a similar but unconfirmed role in the symbiotic digestion of wood. Importantly, our recovery of a largely termite-associated clade of sequences identified as *Lactovum* within the insect-associated *Lactococcus* clade suggests that the genus *Lactococcus* might be polyphyletic [as also observed by Zuo et al. (2018)], and that at least this *Lactovum* clade may be subsumed under *Lactococcus*.

As with *Lactococcus*, the preferential association of members of *Turicibacter* on wood fibers in *O. disjunctus* appears to align with a specialized role in symbiotic digestion. Genomic analysis of *Turicibacter* strains, including those identified from *O. disjunctus* (Ceja-Navarro et al., 2019), possess multiple glycosyl hydrolases that putatively contribute to the lignocellulose digestion. The Carbohydrate Active Enzyme Database (CAZy) reports the genome of *Turicibacter sanguinis* MOL361 to encode around 50 glycosyl hydrolase genes that belonging to 24 different families, including putative glucosidases or xylosidases. The CAZy database also records that the genomes of *Turicibacter bilis* strains ISU324, MMM721, and PIG517 each encode around 30 genes belonging to 12 glycosyl hydrolase families. More relevantly, *Turicibacter* MAGs (g78c1 and g13c1) obtained from the *O. disjunctus* midgut also encode multiple glycosyl hydrolases, a Fe-Mn superoxide dismutase and a Vanillyl-alcohol dioxygenase (Ceja-Navarro et al., 2019), which additionally allows us to speculate their putative roles in lignin oxidation on the surface of wood particles. This metabolic potential of *Turicibacter* strains is also supported by their distribution in the hindguts of another passalid beetle *Veturius* sp. (JGI GOLD study ID:Study ID: Gs0050939), and the termites *Nasutitermes corniger* (where *Turicibacter* forms 17% of the microbiome in the anterior P1 compartment), *Neocapritermes taracua* (11%), and *Termes hospes* (13%; Mikaelyan et al., 2017). Swine-derived *Turicibacter* strains were found to increase in relative abundance and were implicated in lignocellulose decomposition in an anaerobic bioreactor study (Tang et al., 2022), which further supports a degree of ecological specialization in this genus to oxygen-limited environments characterized by lignocellulose degradation. Although a role has not yet been suggested for *Turicibacter* in the termite gut, their conserved association with wood-feeding insects and their preferential enrichment on wood fibers in *O. disjunctus* suggests a long-standing association and adaptation to environments associated with wood digestion. The placement of all *O. disjunctus*-recovered sequences within an insect-restricted clade (distinct from sequences obtained from mammals) of *Turicibacter* supports this idea. Based on the consistent association of *Turicibacter* with wood particles in the AHG of *O. disjunctus*, it is plausible that the termite-associated members also play a hitherto undefined role in polysaccharide degradation.

The enrichment of *Lactococcus* and *Turicibacter* in the fiber-associated community of *O. disjunctus*, combined with published evidence of their metabolic potential, clearly suggests a specialized ecological adaptation for fiber digestion. However, it is imperative to consider the involvement of other taxa, such as the Christensenellaceae R-7 group. Despite their lack of preferential abundance on wood fibers, the presence of the Christensenellaceae R-7 group within the fiber-associated community of *O. disjunctus*, comprising a substantial proportion (approximately 7–10%), indicates their potential contribution to fiber digestion. Notably, our recovery of several insect-restricted clades within the Christensenellaceae R-7 lineage further suggests the existence of host-specific clades that have undergone unique adaptations to thrive in the insect gut environment (Supplementary Figure 3). The wide distribution of Christensenellaceae R-7 members among ruminants, including their presence in the fiber-adherent fraction of the bovine microbiome (Brulc et al., 2009; Daugaliyeva et al., 2022), supports their potential role in anaerobic ecosystems characterized by the degradation of plant material, such as the gut environment of *O. disjunctus*. Although the metabolic potential of Christensenellaceae R-7 members in the gut of *O. disjunctus* remains largely unexplored, their prevalence in other ruminant-associated environments highlights their potential significance in fiber degradation.

In summary, our results highlight the highly compartmentalized nature of cellulose and xylan digestion in the midgut and hindgut of *O. disjunctus*, and that wood particles in the anterior hindgut serve as key surface microhabitats for polysaccharide degradation by a specific fiber-associated microbiome. As with wood-feeding termites and cockroaches, the anaerobic degradation of wood particles is a central process in the complex hindgut ecosystem of passalid beetles. Our study clearly shows that, as in wood-feeding termites (Mikaelyan et al., 2014; Tokuda et al., 2018), wood particles in the AHG of *O. disjunctus* are an important site for cellulose and xylan digestion. The consistent colonization of these wood particles by passalid-restricted phylotypes of *Turicibacter* and *Lactococcus* suggest a degree of habitat endemism and ecological specialization to the beetle’s gut. Phylogenetic analyses of fiber-associated members of *Fibrobacterota* in termites have been shown to broadly reflect host phylogeny (Mikaelyan et al., 2015; Bourguignon et al., 2018). However, only a broader sampling of passalid gut microbiomes can reveal if there are similar patterns of evolution within *Turicibacter* and *Lactococcus* that parallel host phylogeny. Through a comprehensive examination of lignocellulose-feeding insects, including other wood-boring cockroaches (Bauer et al., 2015) and beetles (Schloss et al., 2006; Scully et al., 2013) characterized by complex microbiomes, we can gain valuable insights into the convergent adaptations of bacterial lineages to similar intestinal environments. These thorough investigations have the potential to unravel the intricate relationships between wood-feeding species and their gut microbiomes, propel our understanding of symbiotic wood digestion, and opening up new frontiers for diverse applications.

## Data availability statement

The datasets presented in this study can be found in online repositories. The names of the repository/repositories and accession number(s) can be found below: https://www.ncbi.nlm.nih.gov/genbank/, PRJNA839039 https://www.ncbi.nlm.nih.gov/genbank/, PRJNA914753.

## Author contributions

MS and AM conceived and designed the experiments. MS performed the experiments and analyzed the data. CB-B performed the phylogenetic analysis. MS, CB-B, and AM wrote the manuscript. All authors contributed to the article and approved the submitted version.

## Footnotes

1
http://rsb.info.nih.gov/ij/

2
https://github.com/PacificBiosciences/pbbioconda

3
https://github.com/tseemann/barrnap
