# Supplementary material for: Wood fibers are a crucial microhabitat for cellulose- and xylan- degrading bacteria in the hindgut of the wood-feeding beetle Odontotaenius disjunctus
Source: Front Microbiol. 2023 Jun 28;14:1173696. doi: 10.3389/fmicb.2023.1173696 (PMC10338082; doi:10.3389/fmicb.2023.1173696)
Supplement: Supplementary file 1 [file Data_Sheet_1.docx]

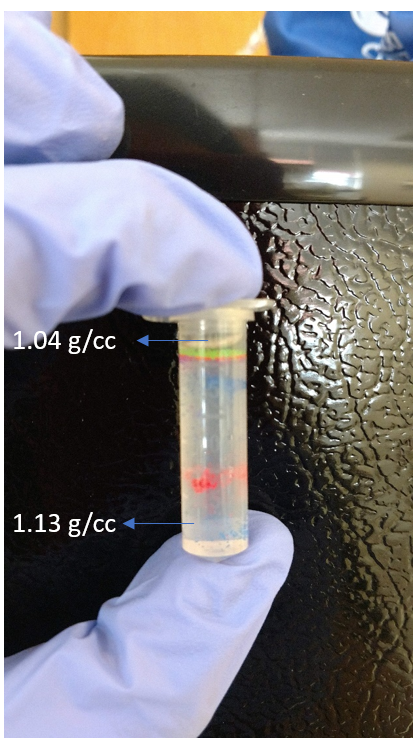


**Supplementary Figure 1.** Percoll density gradient separation of marker beads (of densities 1.02 g/cc, 1.04 g/cc, 1.08 g/cc, 1.09 g/cc, and 1.13 g/cc). The column was prepared by overlaying 30% solution over an equal volume of 70% solution of Percoll, resulting in a discontinuous density gradient. The general location of *O. disjunctus* luminal bands are denoted by arrows with the fiber free fraction being in the vicinity of the 1.04 g/cc beads and the fiber fraction settling in the area of the 1.13 g/cc band
