# Supplementary material for: Wood fibers are a crucial microhabitat for cellulose- and xylan- degrading bacteria in the hindgut of the wood-feeding beetle Odontotaenius disjunctus
Source: Front Microbiol. 2023 Jun 28;14:1173696. doi: 10.3389/fmicb.2023.1173696 (PMC10338082; doi:10.3389/fmicb.2023.1173696)
Supplement: Supplementary file 2 [file Data_Sheet_2.docx]

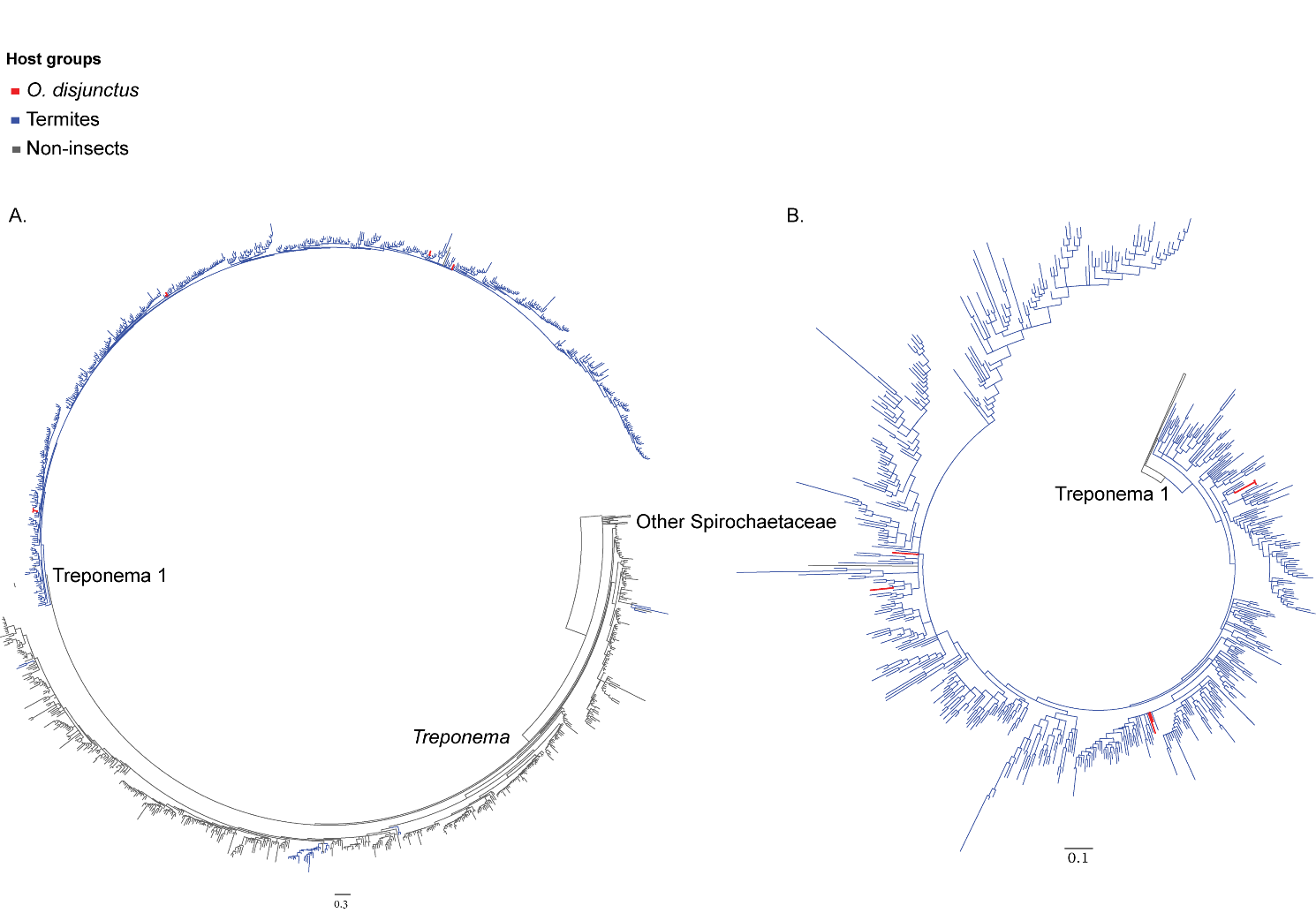


**Supplementary Figure 2.** Consensus tree based on partial 16S rRNA genes for 1000 ultrafastbootstrap replicates of *Treponema* and *Treponema* I (A, sensu Mikaelyan et al. 2015). The subtree (B) highlights the relationships inside *Treponema* I within the broader calculated phylogenetic tree (A). Nodes showing less than 60% support have been collapsed into multifurcations. Edges and circles are respectively colored by diet and taxonomy of the hosts, from which the sequences were obtained.
