# Supplementary material for: Wood fibers are a crucial microhabitat for cellulose- and xylan- degrading bacteria in the hindgut of the wood-feeding beetle Odontotaenius disjunctus
Source: Front Microbiol. 2023 Jun 28;14:1173696. doi: 10.3389/fmicb.2023.1173696 (PMC10338082; doi:10.3389/fmicb.2023.1173696)
Supplement: Supplementary file 3 [file Data_Sheet_3.docx]

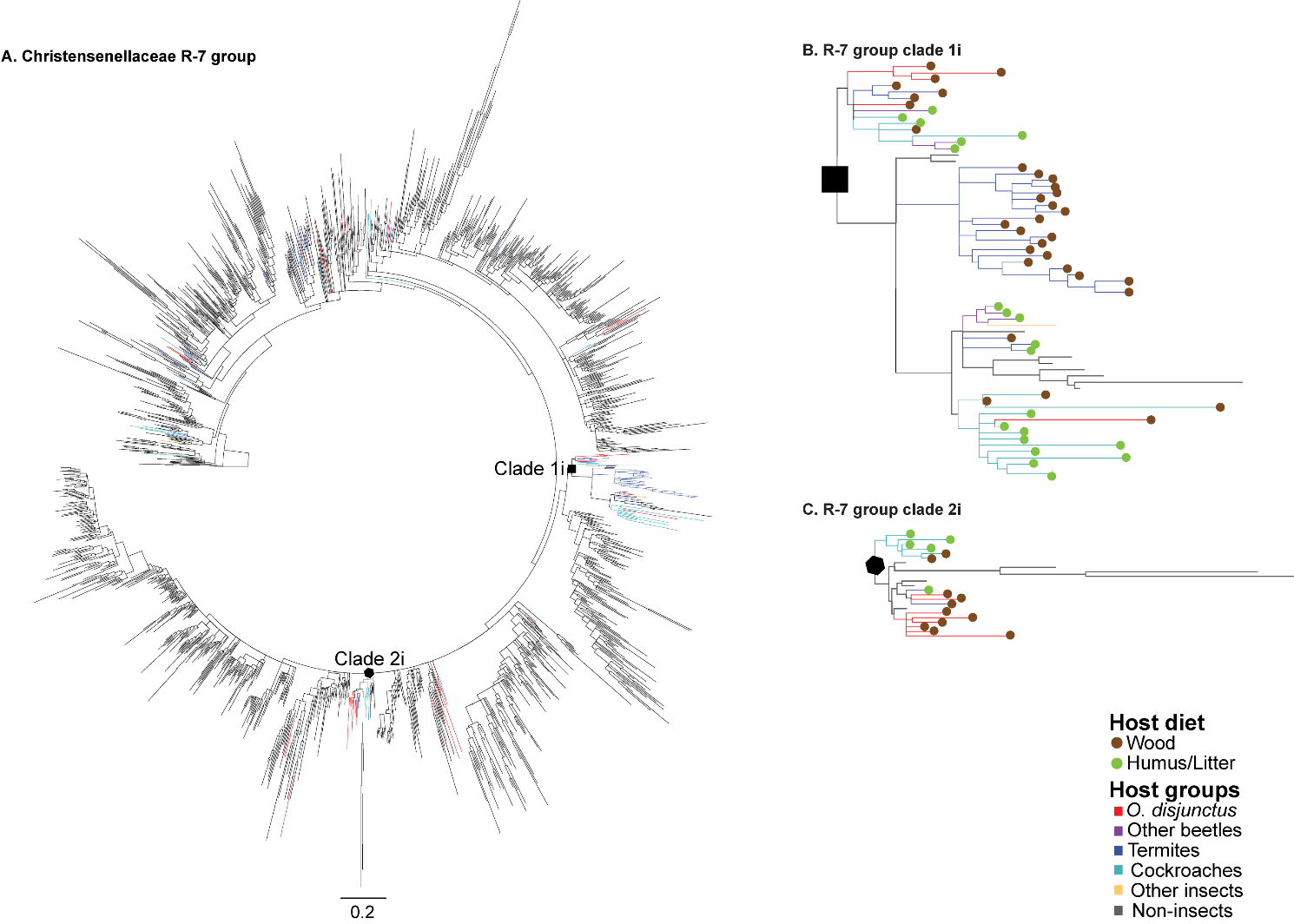


**Supplementary Figure 3.** Consensus tree based on partial 16S rRNA genes for 1000 ultrafastbootstrap replicates of *Christensenellaceae* R-7 group (A). The subtrees (B and C) highlight the relationships inside Clades 1i and 2i within the broader calculated phylogenetic tree (A). Nodes showing less than 60% support have been collapsed into multifurcations. Edges and circles are respectively colored by diet and taxonomy of the hosts, from which the sequences were obtained.
