# Supplementary material for: Wood fibers are a crucial microhabitat for cellulose- and xylan- degrading bacteria in the hindgut of the wood-feeding beetle Odontotaenius disjunctus
Source: Front Microbiol. 2023 Jun 28;14:1173696. doi: 10.3389/fmicb.2023.1173696 (PMC10338082; doi:10.3389/fmicb.2023.1173696)
Supplement: Supplementary file 5 [file Table_1.DOCX]

**Supplementary Table 1** Cellulase and xylanase activity in entire (including the lumen and gut tissue) gut compartments [midgut (MG), anterior hindgut (AHG) and posterior hindgut (PHG)] of *O. disjunctus*. For each assay, activity measured from crude enzyme extracts released by sonication and detergent treatment are reported (in milliunits), along with the total activity (sonication + detergent). One unit is defined as 1 μmol of sugar equivalent released from the substrate per minute, per gram of insect.

| **Enzyme** | **Gut compartments** | **Sonication-associated activity (milliunits)** | **Detergent-associated activity (milliunits)** | **Total activity**  **(milliunits)** |
| --- | --- | --- | --- | --- |
| **Cellulase** | MG | 61.53 ± 35.00 | 7.86 ± 3.50 | 69.4 ± 38.28 |
|  | AHG | 6.98 ± 3.06 | 2.91 ± 1.44 | 9.89 ± 4.46 |
|  | PHG | 3.09 ± 1.34 | 2.22 ± 0.48 | 5.32 ± 1.75 |
| **Xylanase** | MG | 281.29 ± 9.33 | 52.38 ± 1.52 | 333.67± 10.80 |
|  | AHG | 70.42 ± 11.32 | 38.84 ± 3.80 | 109.265 ± 13.20 |
|  | PHG | 46.31 ± 10.78 | 21.82 ± 2.84 | 68.14 ± 11.33 |
