# Supplementary material for: Wood fibers are a crucial microhabitat for cellulose- and xylan- degrading bacteria in the hindgut of the wood-feeding beetle Odontotaenius disjunctus
Source: Front Microbiol. 2023 Jun 28;14:1173696. doi: 10.3389/fmicb.2023.1173696 (PMC10338082; doi:10.3389/fmicb.2023.1173696)
Supplement: Supplementary file 6 [file Table_2.DOCX]

**Supplementary Table 2** The percentage of enzyme (cellulase and xylanase) activity retained in the lumen in the lumen of the midgut (MG), anterior hindgut (AHG) and posterior hindgut (PHG) of *O. disjunctus*. Enzyme activity reported is the sum of measurements from crude enzyme extracts released by sonication and detergent treatment. One unit is defined as 1 μmol of sugar equivalent released from the substrate per minute, per gram of insect.

| **Enzyme** | **Fraction** | **Activity associated with intact tissue (milliunits)** | **Activity associated with luminal content**  **(milliunits)** | **Percentage activity retained in the lumen**  **(%)** |
| --- | --- | --- | --- | --- |
| **Cellulase** | MG | 69.4 ± 38.28 | 14.35 ± 3.26 | 20.6 |
|  | AHG | 9.89 ± 4.46 | 14.41 ± 8.59 | 145 |
|  | PHG | 5.32 ± 1.75 | 10.04 ± 1.19 | 188 |
| **Xylanase** | MG | 333.67± 10.80 | 73.15 ± 4.74 | 21 |
|  | AHG | 109.265 ± 13.20 | 95.47 ± 10.72 | 87 |
|  | PHG | 68.14 ± 11.33 | 78.03 ± 8.66 | 114 |
