# Supplementary material for: Wood fibers are a crucial microhabitat for cellulose- and xylan- degrading bacteria in the hindgut of the wood-feeding beetle Odontotaenius disjunctus
Source: Front Microbiol. 2023 Jun 28;14:1173696. doi: 10.3389/fmicb.2023.1173696 (PMC10338082; doi:10.3389/fmicb.2023.1173696)
Supplement: Supplementary file 7 [file Table_3.DOCX]

**Supplementary Table 3** Cellulase and xylanase activity in luminal fractions from the anterior hindgut (AHG) of *O. disjunctus*, starting with all luminal content, which was further fractionated using density gradient centrifugation into the Fiber-free and Fiber fractions. For each assay, activity measured from crude enzyme extracts released by sonication and detergent treatment are reported (in milliunits), along with the total activity (sonication + detergent). One unit is defined as 1 μmol of sugar equivalent released from the substrate per minute, per gram of insect.

| **Enzyme** | **Fractions** | **Sonication-associated activity (milliunits)** | **Detergent-associated activity (milliunits)** | **Total activity**  **(milliunits)** |
| --- | --- | --- | --- | --- |
| Cellulase | Luminal content | 7.76 ± 5.56 | 6.64 ± 4.40 | 14.42 ± 9.97 |
|  | Fiber-free | 3.51 ± 0.71 | 4.25 ± 0.42 | 7.76 ± 1.03 |
|  | Fiber | 3.35 ± 0.20 | 4.18 ± 0.29 | 7.53 ± 0.37 |
| Xylanase | Luminal content | 37.27 ± 10.1 | 58.2 ± 4.01 | 95.46 ± 10.71 |
|  | Fiber-free | 17.26 ± 0.26 | 24.63 ± 0.51 | 41.90 ± 0.51 |
|  | Fiber | 17.91 ± 0.94 | 25.21 ± 4.19 | 43.13 ± 4.66 |

**Supplementary Table 4 (Excel Sheet)**. Relative abundance of bacterial genera differentially abundant between the fiber and fiber-free community across all samples. Abbreviations: LFC, log2fold change; LFCSE, log2fold Change standard error; padj, adjusted (corrected) p-value.

**Supplementary Table 5 (Excel Sheet).** Relative abundance of bacterial taxa in the Illumina-sequenced amplicon libraries of 16S rRNA genes from the luminal fluid, fiber-free fractions, and fiber fractions. Interactive spreadsheet; classification results can be displayed for different taxonomic levels (1, phylum; 2, class; 3, order; 4, family; 5, genus; 6, OTU defined at 97%)
